# mTOR Signaling in the Inner Ear as Potential Target to Treat Hearing Loss

**DOI:** 10.3390/ijms22126368

**Published:** 2021-06-14

**Authors:** Maurizio Cortada, Soledad Levano, Daniel Bodmer

**Affiliations:** 1 Department of Biomedicine, University of Basel, Hebelstrasse 20, 4031 Basel, Switzerland; maurizio.cortada@unibas.ch (M.C.); s.levano@unibas.ch (S.L.); 2 Clinic for Otorhinolaryngology, Head and Neck Surgery, University of Basel Hospital, Petersgraben 4, 4031 Basel, Switzerland

**Keywords:** cochlea, hair cells, hearing loss, inner ear, mTOR, protection, regeneration

## Abstract

Hearing loss affects many people worldwide and occurs often as a result of age, ototoxic drugs and/or excessive noise exposure. With a growing number of elderly people, the number of people suffering from hearing loss will also increase in the future. Despite the high number of affected people, for most patients there is no curative therapy for hearing loss and hearing aids or cochlea implants remain the only option. Important treatment approaches for hearing loss include the development of regenerative therapies or the inhibition of cell death/promotion of cell survival pathways. The mammalian target of rapamycin (mTOR) pathway is a central regulator of cell growth, is involved in cell survival, and has been shown to be implicated in many age-related diseases. In the inner ear, mTOR signaling has also started to gain attention recently. In this review, we will emphasize recent discoveries of mTOR signaling in the inner ear and discuss implications for possible treatments for hearing restoration.

## 1. Introduction

**Figure 1:**
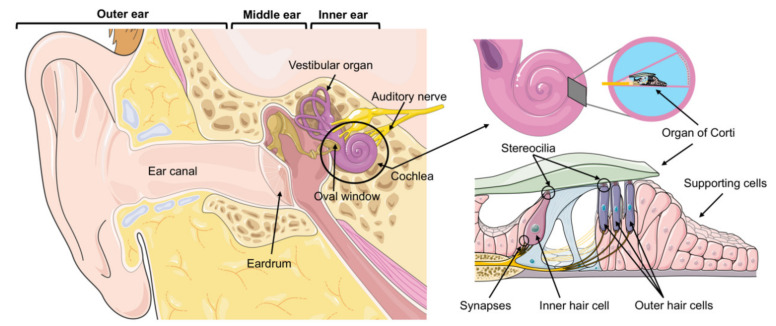
The anatomy of hearing. The spiral-shaped cochlea is the auditory sensory organ where mechanical sound waves are transmitted into electrical signals by the hair cells of the organ of Corti. See the text for further details. The figure was created with Servier Medical Art templates by Servier, which are licensed under a Creative Commons Attribution 3.0 Unported License; https://smart.servier.com (accessed on 16 January 2021).

Sound waves that reach the external ear travel via the auditory canal to the eardrum, where vibrations are transmitted by the ossicles of the middle ear onto the inner ear at the oval window (Figure 1). The inner ear is a fluid-filled space, composed of the vestibular organ for the perception of balance and the spiral cochlea for auditory perception. Sound induced fluid waves in the cochlea lead to a deflection of stereocilia at the top of the hair cells, which are the auditory sensory cells (Figure 1). There are two types of hair cells in the cochlea: one row of inner hair cells (the actual sensory cells) and three rows of outer hair cells, which function as a signal amplifier (Figure 1). Deflection of the stereocilia leads to mechanoelectrical transduction: ion channels open and depolarize the hair cells. Ultimately, the depolarization of the inner hair cells leads to the secretion of glutamate at the inner hair cell synapses and to action potentials which propagate the signal via auditory nerve to the auditory cortex.

Defects at any of these levels, from the outer ear to the auditory cortex, can lead to hearing loss. Hearing loss with its cause in the outer or middle ear is classified as conductive hearing loss. Common causes are mechanical obstruction, infections, or malformations which can be surgically treated. Damage to the inner ear, auditory nerve, central auditory nuclei or cortex is classified as sensorineural hearing loss. Hair cells and their synapses are the most vulnerable structures and are commonly damaged or lost causing sensorineural hearing loss. Many exposures in life can lead to sensorineural hearing loss, such as noise, infections, or ototoxic drugs like aminoglycoside antibiotics and chemotherapeutic agents like cisplatin. Other causes are intrinsic, such as genetic susceptibility or aging. Due to their postmitotic nature, the hair cells do not regenerate upon loss in mammals. Consequently, the hair cells must be protected and preserved life-long.

Sensorineural hearing loss is considered to be the most frequent sensory deficit: According to WHO estimates, there are 466 million persons in the world suffering from disabling hearing loss [1]. One-third of the population ≥65 years is affected by hearing loss [1]. With the increase in life expectancy and increasing number of elderly people in the population, the number of affected individuals will also increase. It is expected that by 2050, there will be over 900 million people suffering from hearing loss [1]. Despite these growing numbers, there are currently no curative therapies for sensorineural hearing loss. Current treatment options consist of hearing aids or cochlea implants for severely affected patients. In order to be able to cure hearing loss in the future, different strategies have been proposed: A potential cure for genetic forms of hearing loss could be gene therapy [2,3]. Another promising strategy is the use of stem cell or molecular therapies to restore the lost hair cells, and therefore treat hearing loss from various causes [2,3,4]. A further approach include pharmacological interventions which either inhibit cell death or promote cell survival pathways in sensory hair cells [3,4].

The mechanistic or mammalian target of rapamycin (mTOR) is a serine/threonine kinase which, as part of two multi-protein complexes, is a central cellular hub governing cell growth and cell survival among others [5]. It is an evolutionary conserved kinase, and its involvement in cancer, metabolic diseases, neurological and neurodegenerative diseases underlines its biological importance [6]. The drug rapamycin, which inhibits mTOR signaling led to the discovery of the *TOR* genes [7]. Rapamycin was the first drug for which a life-prolonging effect was shown in mammals [8]. Accordingly, the mTOR signaling pathway has been shown to play an important role in aging and age-related diseases [9]. Recently, mTOR signaling has also started to gain attention in the inner ear. In this review, we will highlight recent findings and discuss potential roles for hearing restoration.

## 2. mTOR Signaling

The mTOR kinase exists in in two distinct multi-protein complexes, which differ in composition, structure and function. They lie at the center of the mTOR signaling pathway and regulate distinct targets to exert their functions. In this section, we will introduce the major components of the mTOR signaling network. For deeper understanding of this pathway, we refer the readers to recent reviews [10,11,12].

### 2.1. mTORC1

The mTOR complex 1 (mTORC1) is the better studied of both complexes. Structurally, the complex is a homodimer composed of mTOR, the defining and essential subunit regulatory-associated protein of TOR (RAPTOR) and mammalian lethal with SEC13 protein 8 (mLST8) [13,14,15,16]. The mTORC1 is rapamycin sensitive: rapamycin inhibits mTORC1 after building a complex with FK506-binding protein of 12 KDa (FKBP12) [17,18], possibly by blocking the access for substrates to the catalytic site [12]. Substrate recruitment by mTORC1 is accomplished in part via binding of RAPTOR to a TOR signaling (TOS) motif [12,19,20].

Upstream activation of mTORC1 is regulated in form of an ‘AND’ gate [10], in that only when both growth factors and nutrients are available mTORC1 is activated. Upon ligand binding to receptor tyrosine kinases (RTKs) or G-coupled receptors (GPCRs), the receptors activate class I phosphoinositide 3-kinase (PI3K) family members which produce phosphatidylinositol (3,4,5)-tris- phosphate (PIP3) [21,22]. Inactive AKT is recruited to membranes by binding to PIP3 and is phosphorylated at Thr308 and Ser473 by phosphoinositide-dependent protein kinase 1 (PDK1) and mTOR complex 2 (mTORC2), respectively. This leads to AKT activation. Activated AKT inhibits the tuberous sclerosis complex (TSC) [23,24,25], which ends TSC-mediated inhibition of the RAS homolog enriched in brain (RHEB), finally activating mTORC1 [21,22]. The functional TSC consists of TSC1, TSC2, and Tre2-Bub2-Cdc16 (TBC) 1 domain family, member 7 (TBC1D7) [26]. In insulin/ insulin-like growth factor-1 (IGF-1) signaling, TSC2 is directly phosphorylated by AKT, which leads to the dissociation of the TSC from the lysosome [27] and disrupts the binding of TSC2 to TSC1 [23]. TSC2 has a guanosine triphosphatase (GTPase)-activating protein (GAP) activity towards RHEB [28]. Thereby, TSC stimulates the GTP hydrolysis of RHEB, converting this protein into an inactive GDP-loaded RHEB resulting in a decrease in mTORC1 activity [28]. With TSC inhibited by AKT, the active GTP-bound RHEB can activate mTOR on the lysosome [10,29,30]. In order for mTORC1 to be activated by RHEB, mTORC1 it needs to be recruited from the cytoplasm to the lysosome and this depends on the availability of amino acids, glucose and other nutrients [10]. Central to amino acid sensing are the Ras-related family of small GTPases (RAGs) [31,32], which localize to the lysosome via the RAGULATOR complex [33,34]. In response to amino acids, mTORC1 translocates to the lysosomal membrane and via RAPTOR directly binds to RAG–RAGULATOR complex [33,35]. The GAP activity towards the RAGs 1 (GATOR1) complex has a GAP activity to RAGs and therefore inhibits mTORC1 activity [12,36,37]. The GATOR1 complex itself is negatively regulated by GATOR2 [36]. The KICSTOR complex binds and translocates GATOR1 to the lysosomal surface and negatively regulates mTORC1 [38,39]. SESTRIN-2 has been discovered as the leucine sensor to the mTOR pathway [40]. SESTRIN-2 binds and blocks GATOR2 when leucine levels are low, whereas the binding of leucine to SESTRIN-2 blocks this interaction [40]. In arginine-depleted conditions, the cellular arginine sensor for mTORC1 (CASTOR1) binds and inhibits GATOR2 [41]. Folliculin (FLCN) is another amino acid sensor interacting directly with the RAGs [42,43]. The S-adenosylmethionine sensor upstream of mTORC1 (SAMTOR) binds to and promotes the mTORC1 inhibiting function of GATOR1 and KICSTOR [44]. This complex can be disrupted by S-adenosylmethionine (SAM) which is sensitive to methionine levels [44]. On the lysosome, there are additional proteins acting as amino acid sensors: the lysosomal vacuolar ATPase (v-ATPase) [45] and Solute carrier family 38 member 9 (SLC38A9) [46]. Amino acids are the best-known nutrients to activate mTORC1. However, apart from amino acids, other nutrients also activate mTORC1 [10,34]. The AMP-activated protein kinase (AMPK) senses cellular energy status and interacts in multiple ways with mTORC1 to inhibit its activity [12]. Interestingly, reactive oxygen species (ROS) have also been shown to either activate or inactivate mTORC1 [47,48,49] and mTORC1 has been shown to be redox sensitive [50,51,52].

Despite the important role mTORC1 plays in cells, there are not many direct downstream targets that have been identified to date. The most known direct targets are the ribosomal S6 kinase (S6K), 4E binding protein 1 (4E-BP) and UNC-51-like kinase 1 (ULK1) [53]. In addition, mTORC1 also acts via transcription factors such as transcription factor EB (TFEB) [54,55,56], activating transcription factor 4 (ATF4) [57] or hypoxia inducible factor 1α (HIF1α) [10,58]. The general purpose of mTORC1 is to promote anabolic metabolism (protein, lipid and nucleotide synthesis) and to block the degrading catabolic processes (autophagy) [53]. mTORC1 promotes protein synthesis by activating and phosphorylating S6K on Thr389 and by an inhibitory phosphorylation on 4E-BP [10,12,53,59,60]. mTORC1 stimulates lipid synthesis via S6K or by inhibiting LIPIN1, which in turn activate sterol-regulatory element-binding protein (SREBP) [53,58,61]. Nucleotide synthesis is promoted by mTORC1 via ATF4 [57] or via S6K which further activates carbamoyl–phosphate synthetase 2, aspartate transcarbamoylase, dihydroorotase (CAD) enzyme [62,63]. mTORC1 also promotes glycolysis via HIF1α and the pentose phosphate pathway via SREBP [58]. Interestingly, mTOR also enhances mitochondrial biogenesis via 4E-BP [64,65] or via transcription factor yin-yang 1 (YY1) and peroxisome-proliferator-activated receptor coactivator (PGC)-1α coactivation [66]. To suppress catabolic processes, mTORC1 inhibits autophagy via inhibitory phosphorylations of ULK1 and autophagy-related gene (ATG) 13 protein [67,68,69,70]. mTORC1 also inhibits catabolism by phosphorylating and inhibiting TFEB, which promotes lysosomal biogenesis [54,55,56]. Table 1 summarizes the major mTORC1 signaling components.

### 2.2. mTORC2

In contrast to mTORC1, mTORC2 is rapamycin insensitive [74], however, it has also been shown to be inhibited by prolonged treatment with rapamycin [75]. mTORC2 is composed of mTOR, mLST8 [15], the rapamycin-insensitive companion of mTOR (RICTOR) [74,76] and MAPK-interacting protein 1 (mSIN1) [77,78,79]. mTORC2 structure resembles that of mTORC1 and exists in a homodimeric complex [80,81,82].

Growth factors are the best-known activators of mTORC2. Similarly to the activation of mTORC1, growth factors activate mTORC2 via the PI3K pathway, however, the precise mechanism remains elusive [10,11]. mSIN1 contains a pleckstrin homology (PH) domain and it has been reported that this domain blocks mTORC2 activity, whereas PI3K activation releases this inhibition [83,84]. An alternative model is that mTORC2 lies permanently at the plasma membrane and is activated by AKT [11]. In addition to growth factors, mTORC2 has also been shown to be activated by small GTPases such as oncogenic Ras [85]; or by other signaling pathways, such as the Hippo pathway [86], the Wnt pathway [87] and its non-canonical planar cell polarity (PCP) pathway [88], and it is activated by AMPK [89] (all discussed in [11]). mTORC2 exists at different subcellular localizations, which might be differentially regulated [11]. Interestingly, an important pool seems to be located at the mitochondria and the mitochondria-associated endoplasmic reticulum (ER) membrane (MAM) (reviewed in [11,90]).

The main effectors and downstream targets of mTORC2 are protein kinase A, C and G (AGC) family members [91]. The most studied target is AKT (protein kinase B, PKB) phosphorylated on Ser473 [92,93]. Furthermore, mTORC2 also phosphorylates AKT on Thr450 [94]. Other AGC family members regulated by mTORC2 are protein kinase C (PKC) [15,74,76] and serum- and glucocorticoid-induced protein kinase 1 (SGK1) [95]. Interestingly, mTORC2 has also been shown to phosphorylate and inhibit the cystine-glutamate antiporter xCT, which is also known as Solute Carrier Family 7 Member 11 (SLC7A11) [96]. Table 2 summarizes the major mTORC2 signaling components.

### 2.3. Feedback Mechanisms

There are also negative feedback loops between mTORC1 and mTORC2, which have been discussed as possible reasons for the lack of efficiency of rapamycin and its analogues to treat cancer [18]. mTORC1 has been shown to downregulate and inhibit the insulin receptor substrates (IRS) via S6K [97,98,99], therefore, negatively regulating insulin-PI3K-AKT and mTORC2 activation. Another feedback mechanism functions via growth factor bound-receptor protein 10 (GRB10): mTORC1 phosphorylates and stabilizes GRB10, which binds the insulin receptor and negatively regulates insulin-PI3K-mTORC2 signaling [100,101,102].

## 3. mTOR Signaling in Auditory Sensory Hair Cell Regeneration

It has long been postulated that the damage and death of the auditory sensory hair cells is the main underlying cause of hearing loss [103]. The functional principle of cochlea implants is also based on this notion, where implanted electrodes bypass the hair cells and stimulate the auditory nerve directly [104]. Studies by Kujawa and Liberman, however, have shown that especially in age-related and noise-induced hearing loss, an early loss of the hair cell innervating synapses occurs even before or without hair cell loss; a phenomenon named cochlear synaptopathy [103,105,106].

It was long assumed that the postmitotic hair cells cannot regenerate upon loss [107]. However, since the discovery of hair cell regeneration in birds [108,109,110,111], a potential regenerative therapy to replace lost hair cells has come to the attention of researchers. This regeneration in birds emanates from the supporting cells that surround the hair cells in the organ of Corti (Figure 1). The mechanisms proposed are either mitotic regeneration or direct trans-differentiation of supporting cells to hair cells [112]. In contrast to birds, adult mammalian hair cells do not spontaneously regenerate once lost, and the loss of synapses in cochlear synaptopathy has also been shown to be mainly permanent [103]. Interestingly, supporting cells isolated from the cochlea of neonatal mice retain the ability to produce hair cells [113] and hair cell regeneration has been shown to some extent in the vestibular epithelium of mammals [114,115]. However, in the adult mammalian cochlea, lost hair cells are not regenerated.

An important approach to design hair cell regeneration therapies has been to study and exploit the signaling pathways and transcription factors involved in the development and maturation of hair cells [112]. Based on this rationale, a few compounds to treat hearing loss by regenerating lost hair cells are being tested in clinical trials: an adenoviral atonal homologue 1 (*ATOH1*, also known as *Math1*) gene therapy has been tested to treat hearing loss, but no major improvement in hearing or vestibular function has been found [116]. *Atoh1* has been shown to be sufficient and necessary for auditory hair cell differentiation [117], and overexpression produces extra hair cells [118]. Other clinical studies use pharmacological approaches to induce hair cell regeneration: a NOTCH inhibitor [119] or the combination of a glycogen synthase kinase-3 (GSK3) inhibitor together with valproic acid, an antiepileptic drug [120], are being tested to treat sensorineural hearing loss. Unfortunately, no regenerative therapy has made it to the clinic to date.

Two early studies using rapamycin suggested the involvement of mTOR signaling in the proliferation of avian and mammalian sensory epithelia [121,122]. However, the role of mTOR signaling in cochlear hair cell regeneration has only recently started to be elucidated. Zheng-Yi Chen and colleagues have shown that the proliferation and regeneration of hair cells is indeed possible in the adult cochlea, both *in vivo* and *in vitro* [123]. By co-activating both *c-myc* (*Myc*) and *Notch1* genes, they were able to induce the proliferation of both supporting cells and inner hair cells in adult cochleae [123]. Only when *Myc* and *Notch1* were transiently activated in the cochlea, the supporting cells were able to respond to *Atoh1* overexpression and trans-differentiate into hair cell-like cells [123]. These regenerated hair cells had functional transduction channels and even made contact with surrounding neurites [123]. Most interestingly, the authors detected strong labelling of the phosphorylated ribosomal S6 protein in newly proliferating supporting cells, which lies downstream of S6K and mTORC1. Moreover, rapamycin reduced then number of proliferating cells and the number of regenerated hair cell-like cells. By using MHY1485, a small molecule known to inhibit autophagy and activate mTOR [124], they also showed that mTOR partially compensates *Myc* function. Thus, the authors concluded that the mTOR pathway is involved in the proliferation and regeneration of cochlear hair cells [123].

Recent work by Li and Doetzlhofer has shown that the RNA binding protein LIN28B promotes the proliferation capacity and reprogramming of supporting cells to generate hair cells via the AKT-mTORC1 pathway [125]. The intrinsic ability of cochlear supporting cells to (re)generate hair cells is quickly lost during the first postnatal days. Already epithelial cells of postnatal day (P)5 mice fail to expand and produce hair cells [125]. Using both an organoid culture system and cochlear explant cultures from P5 transgenic mice overexpressing *Lin28b*, Li and Doetzlhofer showed that organoids formed an increased number of hair cell clusters than the control organoids. The overexpression of *Lin28b* in organoids also stimulated the de-differentiation of supporting cells into prosensory-like cells with greater capabilities to generate hair cells. Overexpressing *Lin28b* in cochlear explants moreover increased the production of new hair cells by nonmitotic mechanisms. In contrast, the deletion of *Lin28a/b* or the overexpression of their inhibitor *let-7* miRNA decreased the hair cell generation capabilities of supporting cells [125]. Most interestingly, both the phosphorylation of the S6 protein at Ser240/244 and AKT phosphorylation at Ser473 decreased during postnatal development in cochlear explants (in parallel with the decline in hair cell generation capabilities) [125]. Accordingly, the deletion of *Lin28a/b* or *let-7* miRNA overexpression decreased the phosphorylation levels of AKT, S6, and 4E-BP in neonatal mice cochleae and organoids. In contrast, higher phosphorylation levels of AKT and S6 have been detected in *Lin28b* overexpressing organoids than in control organoids [125]. Notably, rapamycin attenuated supporting cell proliferation and hair cell generation promoted by *Lin28b* overexpression in organoids and cochlear explants. These findings suggest that LIN28B controls supporting cell plasticity via the activation of mTOR signaling [125]. In summary, these recent studies indicate an important role of mTOR in hair cell regeneration.

Interestingly, a recent study has also shown that the disruption of mTOR function by rapamycin in neonatal mice leads to hearing loss [126]. Postnatal mice treated with rapamycin presented reduced ribbon synapse numbers and impaired exocytosis in ribbon synapses of inner hair cells. Adult mice at P28 treated with rapamycin presented normal cochlear morphology and hearing function [126]. These results indicate that mTOR signaling might also play an important role in the postnatal development and formation of cochlear hair cell synapses.

An inner ear specific conditional deletion of the phosphatase and tensin homolog (*Pten*), key modulator of PI3K, induced AKT hyperactivation and modulated the activation of GSK3β during the embryonal stage [127]. These *Pten* deficient mice presented neuronal abnormalities and additional rows of cochlear hair cells [127]. Therefore, PTEN/PI3K-AKT signaling might also be involved in inner ear development.

**Figure 2:**
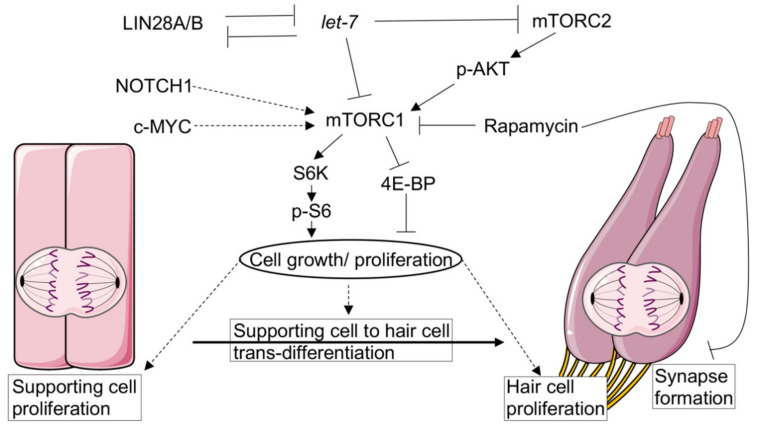
mTOR signaling in auditory sensory hair cell regeneration. mTORC1 is involved in supporting cell proliferation and hair cell (re)generation, effects that can be inhibited by rapamycin. Moreover, rapamycin inhibits inner hair cell synapse maturation at postnatal ages. mTORC2 might also be involved in hair cell generation, since p-AKT decreases when hair cell generation is lost but increases again when the regenerative capacity is regained. Dashed lines indicate unknown mechanisms. The figure was created with Servier Medical Art templates by Servier, which are licensed under a Creative Commons Attribution 3.0 Unported License; https://smart.servier.com (accessed on 16 January 2021).

mTOR signaling regulates many essential cell processes; while mTORC1 regulates cell growth and metabolism, mTORC2 controls cell survival and cytoskeletal rearrangement. Recent studies indicate the involvement of mTOR signaling in auditory sensory hair cell regeneration, however, the precise mechanisms remain unknown (Figure 2). The reactivation time point of mTOR-dependent and mTOR-independent proteins seems to be critical for the development of sensory cells in the inner ear and maintenance of their regenerative capacities. Therefore, modulating tissue-specific mTOR, albeit a challenge, might also be an interesting target for regenerative medicine to treat hearing loss.

## 4. mTOR Signaling in Auditory Sensory Hair Cell Survival and Death

Prevention is the most effective strategy against hearing loss. Therefore, protecting and preserving sensible structures of the inner ear are crucial for hearing. Consequently, interventions to promote hair cell (and synapse) survival pathways or to inhibit hair cell death pathways are important therapeutic strategies. Oxidative damage is an important contributor to hearing loss. Thus, many antioxidants or stress/death signaling inhibitors have been tested to treat hearing loss in clinical trials [3,128]. However, none have made a breakthrough to date for the treatment of hearing loss and established in the clinic.

The protective role of rapamycin against damage to the inner ear has already been shown in numerous *in vitro* and *in vivo* studies. Several *in vivo* studies have reported that rapamycin protected hair cells and hearing function against the ototoxic effects of the chemotherapeutic agent cisplatin [129], the aminoglycoside antibiotic gentamicin [130], or against noise-induced hearing loss [131]. Moreover, rapamycin has been shown to protect against age-related hearing loss [132,133] and age-related outer hair cell loss [134]. Rapamycin has not only protective effects against hair cell damage [135], but also against the degeneration of the spiral ganglion (neurons innervating the sensory hair cells) [136] caused by aminoglycoside antibiotics *in vitro*. A further study using the rapamycin analog temsirolimus has also shown protection against spiral ganglion neuron degeneration *in vivo* [137]. In line with the protective effects of rapamycin, mTORC1 signaling has shown to be activated after damage to the cochlea: the activation of S6K and/or its downstream target S6 has been shown after damaging the cochlear sensory epithelium with gentamicin [138,139] or with cisplatin [140] and in the cochlea of aged mice [132]. Further supporting evidence that mTORC1 overactivation is involved in hair cell damaging events in the inner ear comes from a study where the conditional deletion of *Raptor* in the neurosensory epithelium protected mice against age-related hearing loss, both by preventing hair cell and synapse loss [132]. Conversely, *Tsc1* conditional deletion in the neurosensory epithelium was accompanied by a sustained activation of mTORC1 and resulted in an early onset age-related hearing loss—while rapamycin treatment ameliorated this effect [132]. The authors used a *Atoh1*-Cre mouse for the conditional deletion of *Raptor* and *Tsc1*, which leads to Cre-mediated recombination in the cochlea as early as on embryonic day 14.5 (in addition to further regions such as the hindbrain, spinal cord and intestine) [141]. At this embryonic stage, hair cells are beginning to differentiate in the cochlea [141]. Despite this early induction of the knock-out, the authors did not observe any developmental defects in both *Raptor* or *Tsc1* conditional knock-out mice [132]. The authors did also not find an upregulation of p-AKT Ser473 after the conditional deletion of *Raptor* [132], an effect commonly seen due to the negative feedback of mTORC1 towards IRS-PI3K-AKT (see Section 2.3).

Oxidative stress is known to modulate mTORC1 signaling [49]. The *Tsc1* conditional knock-out mice had elevated ROS markers and a disbalance of pro-oxidant/antioxidant gene expression levels [132]. Treatment with the antioxidant N-acetylcysteine (NAC) strongly lowered p-S6 levels and rescued hair cell loss [132]. This suggests that elevated ROS levels and oxidative stress are hair cell-damaging events in *Tsc1* knock-out mice. Mitochondria are known as important sources of ROS, however, results from transmission electron microscopy (TEM) indicated no mitochondrial defects in hair cells of *Tsc1* conditional knock-out mice. In contrast, the authors detected abnormal peroxisomes by TEM in hair cells and observed TSC1 in peroxisomes of hair cells [132]. Based on these findings, the authors suggested the peroxisome as a principal source of ROS and as regulating organelle of mTORC1 signaling in the auditory hair cells [132].

An important downstream event of mTORC1 is the regulation and inhibition of autophagy (see Section 2.1). The importance of autophagy in the development of the cochlea and in different hearing loss models has been recognized (reviewed in [142], see [126]). Fu et al. showed that, after exposure with the aminoglycoside neomycin, *Tsc1* conditional knock-out mice had much less GFP-LC3 puncta and no autophagosomes compared to control mice. Therefore, autophagy was impaired in *Tsc1* knock-out mice [132]. Mice deficient in *Atg5* in hair cells showed a normal development of hair cells, however, with rapid postnatal degeneration of the hair cells and profound hearing loss already occurring at hearing onset (P14) [143]. Mice with a conditional deletion of *Atg7* specifically in outer hair cells also showed profound hearing loss, due to damage to the stereocilia, the degeneration of outer hair cell afferent synapses and eventually outer hair cell loss. Loss of *Atg7* implicated impaired autophagy and dysfunction of mitochondria, leading to the accumulation of damaged mitochondria [144]. Moreover, impaired autophagy correlated with both noise-induced hearing loss [131] and aminoglycoside-induced hair cell loss severity [135]. Induction of autophagy protected against hair cell loss in both studies, whereas the inhibition of autophagy increased hair cell loss leading to hearing loss [131,135].

AMPK, which interacts in multiple manners with mTOR [12], has also been shown to modulate hearing loss. A study reported that treatment with the AMPK activator AICAR increases PGC-1α mRNA levels and that mice treated with AICAR recover faster from noise insults [145]. *Ampkα1* knock-out mice and wildtype mice had similar hearing thresholds before and directly after noise exposure [146]. However, seven days after noise exposure, *Ampkα1* knock-out mice had significantly higher thresholds than wild-type mice [146]. These results indicate disrupted recovery from noise-insult due to *Ampk* loss. The authors showed that this effect was at least in part due to the reduced expression of BK channels, suggesting that AMPK might stimulate BK channels and prevent noise-induced Ca2+-overload [146]. In contrast, it has also been shown that noise-activated AMPKα by phosphorylating AMPKα at Thr172, and that phosphorylation levels augment with increasing noise levels [147]. The knock-down of *Ampkα1* or its activating liver kinase B1 (*Lkb1*) by siRNA or pharmacological inhibition with Compound C protected the mice against noise-induced hearing loss, and preserved hair cells and synaptic ribbons from degeneration [147]. Interestingly, reducing the AMPKα1 activity rescued premature hearing loss in a mouse model of mitochondrial deafness [148]. These mice showed significantly less cell death, synapse and spiral ganglion neuron loss [149]. Single heterozygous *Ampkα1* knock-out mice also completely recovered their hearing thresholds from a noise insult from which the wild-type mice only partly recovered [149]. These results reveal a contradictory role of AMPK in hearing loss. Given that AMPK inhibits mTORC1 (see Section 2.1 and [12]), and that it phosphorylates ULK1 at different sites to promote autophagy [70,150,151], we would expect a protective effect on hearing loss. Although the reason for these conflicting results is not clear, we can note that the complete deletion of *Ampkα1* has deleterious effects [146], whereas partial inhibition by siRNA [147] or heterozygous knock-out [148,149] has protective effects on hearing loss. AMPK might be protective, but the increasing levels after noise exposure can tip the balance towards hair cell loss [147]. Moreover, it is important to note that there was no difference in ULK1 phosphorylation on the AMPK-dependent site Ser555 between wild-type mice and *Ampkα1* heterozygous knock-out mice [149]. Phosphorylation of mTOR on Ser2448 (AKT and/or S6K depend site of unknown significance, see [152]) was also unchanged between wild-type mice and *Ampkα1* heterozygous knock-out mice [149]. These results suggest that deleterious effects mediated by AMPK are independent of autophagy and mTOR.

The antidiabetic drug metformin has also been shown to inhibit mTORC1 signaling, via mechanisms dependent on AMPK and the TSC at lower doses and AMPK/TSC independent mechanisms at higher doses [153]. In the inner ear, metformin has shown protective properties. It protected against gentamicin [154] and cisplatin [155,156] ototoxicity in an auditory cell line and against gentamicin-induced hair cell death in cochlear explants *in vitro* [157,158]. Metformin was also otoprotective *in vivo* against noise-induced hearing loss [159], cisplatin-induced hearing loss [156], pneumococcal meningitis-induced hearing loss [160], but not against gentamicin-induced hearing loss in guinea pigs [158].

Interestingly, in a model of Pendred syndrome, a syndromic form of hereditary hearing loss, cochlear epithelial cells derived from patient-derived induced pluripotent stem cells showed that both rapamycin and metformin are protective against cell death [161]. In a follow-up study, the authors defined a low-dose rapamycin treatment for Pendred syndrome [162] which is now being investigated in a clinical trial [163].

SESTRIN-2 is one of the upstream modulators of mTORC1 and can exert its function via AMPK during energy sensing and via the inhibition of GATOR2 during amino-acid sensing [164]. SESTRIN-2 as a leucine sensor inhibits mTORC1 activation when leucine levels are low [40] (see Section 2.1). The roles of Sestrins in the inner ear have only recently started to be elucidated. *Sestrin-2* is expressed in hair cells, supporting cells and the spiral ganglion neuron in the cochlea [138,165]. SESTRIN-2 levels in the cochlea are both reduced in aged-mice or after damage with gentamicin [138,165]. Notably, the ablation of *Sestrin-2* enhanced both age-related hearing loss [165] and gentamicin-induced hair cell loss [138]. A potential role of mTORC1 overactivation in the increased susceptibility of *Sestrin-2* knock-out mice to ototoxic damage of hair cells has been discussed [166]. In addition to regulating mTORC1, Sestrins are implicated in different cellular processes and are considered to be essential components of the antioxidant defense mechanism.

**Figure 3:**
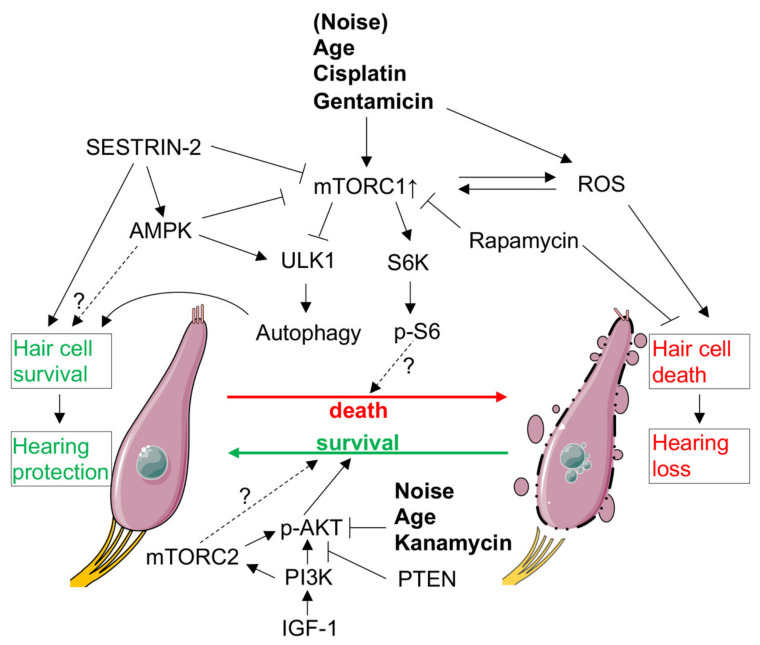
mTOR signaling in auditory sensory hair cell survival and death. mTORC1 is activated after damage due to age or ototoxic drugs which leads to hair cell death as well as damage to the spiral ganglion neurons/synapses. Rapamycin protects against these damaging insults by preserving the hair cells and related neural structures. The downstream damaging effects of mTORC1 overactivation are not well known. These might be due to ROS and the inhibition of autophagy. Although PI3K-AKT signaling protects against hair cell death, the role of mTORC2 remains unknown. Dashed lines indicate unknown mechanisms or effects. The figure was created with Servier Medical Art templates by Servier, which are licensed under a Creative Commons Attribution 3.0 Unported License; https://smart.servier.com (accessed on 16 January 2021).

In contrast to mTORC1 signaling, the role of mTORC2 in the inner ear remains unknown to date (Figure 3). Nevertheless, there is important evidence that the mTORC2 regulating PI3K-AKT pathway is associated with hair cell protection in the inner ear. Early studies using aminoglycosides found that mice treated with kanamycin for seven days showed a downregulation of PI3K-AKT signaling in hair cells [167]. Neonatal rat cochlear explants exposed to PI3K or AKT inhibitors together with gentamicin exhibited increased hair cell damage in comparison to gentamicin alone [168]. Similarly, AKT signaling was reduced in the hair cells of aged mice [169]. Interestingly, mice exposed to mild noise-insults fully recovered their hearing with no change of p-AKT Ser473 in hair cells, whereas detrimental noise-insults led to permanent hearing loss and the concomitant downregulation of p-AKT Ser473 in hair cells [170]. This study also showed that knock-down of the regulatory PI3K subunit p85α reduced p-AKT Ser473 levels and led to permanent hearing loss after mild noise-insults, while control mice fully recovered [170]. Moreover, *Akt1*-knockout mice recovered less fast from mild noise-insults than wild-type mice [170]. These results indicate that PI3K-AKT signaling confers protection against damage to the inner ear and is essential for hair cell survival. Another study used immortalized multipotent otic progenitor cells cultured in suspension (otospheres) and investigated signaling pathways involved in proliferation versus differentiation to identify the mechanisms involved in hair cell survival [171]. They used an unbiased RNA-seq approach to investigate the transcriptome of proliferating versus differentiating otospheres, and found that the PI3K-AKT and mTOR pathway were top pathways of differentially expressed genes [171]. For their subsequent analyses, the authors focused on PI3K signaling. After the pharmacological inhibition or conditional deletion of *Pten* (which antagonizes PI3K activation), the authors showed increased hair cell survival after gentamicin-induced damage in cochlear explants [171]. In addition, AKT has shown to be important for normal hearing, since *Akt1* and *Akt2/3* double knock-out mice showed elevated hearing thresholds [172]. IGF-1, which activates PI3K-AKT signaling via its receptor (see Section 2.1), has shown to be important in both development and cochlear protection (reviewed in [173] and [174]). IGF-1 was protective in animal models of noise-induced hearing loss [175] and aminoglycoside-induced hair cell loss [176]. Recently, IGF-1 also showed involvement in the preservation of cochlear ribbon synapses [177]. Moreover, different *IGF1* gene mutations in humans are associated with hearing loss [174]. The therapeutic effect of IGF-1 in humans has also been investigated in a randomized controlled clinical trial in patients with sudden sensorineural hearing loss refractory to systemic corticosteroid treatment (compared to intratympanic dexamethasone injections). Although there was no difference in the primary outcome (hearing improvement in pure-tone audiometry average hearing thresholds 8 weeks after treatment), there was a significant improvement in the IGF-1-treated group in pure-tone average hearing thresholds occurring over time [178]. Based on all these results, the question arises whether mTORC2 signaling could exert a protective role in the inner ear (Figure 3).

## 5. Open Questions

Despite recent advancements in the understanding of mTOR signaling in the inner ear, there are many open questions that remain. The most advanced study that investigated the role of mTORC1 in hearing loss is the study performed by Fu et. al. [132]. They generated mouse models with deletions of *Raptor* and *Tsc1* in hair cells and supporting cells [132]. Due to the little amount of hair cells in the inner ear, many studies investigate the entire sensory epithelium (hair cells together with supporting cells) instead of specific cell types. An important question that needs to be addressed is the cell specificity of mTORC1 role in the inner ear: is mTORC1 deleterious in the hair cells, or is overactivation of mTORC1 in the supporting cells important for the damaging effects? What is the role of mTORC1 in the spiral ganglion neurons? Different cell types might have divergent mTOR effects. Moreover, to understand what makes mTORC1 overactivation a damaging event in hair cells will need to scrutinize the downstream mechanisms. Production of high levels of ROS and the inhibition of autophagy are common mechanisms in ototoxicity and modulated by mTORC1. Further investigations to clarify the oxidative function of mTORC1 in the inner ear compartments are required. The interplay between upstream modulators such as Sestrins and AMPK with mTORC1 will also need further investigation in the inner ear.

The role of mTORC2 in the inner ear is unknown. Rapamycin is known to be a mTORC1-specific inhibitor, but prolonged rapamycin treatment has also been shown to inhibit mTORC2 [75]. Rapamycin was protective against hair cell and hearing loss in numerous studies, even over a prolonged period of time. Therefore, potential effects on mTORC2 also need to be considered. Although much evidence points to a protective role on hair cells from the PI3K-AKT pathway, the direct involvement of mTORC2 remains to be demonstrated.

In addition to investigating to the role of mTOR under pathological conditions (age, noise, ototoxic agents, etc.), it will also be interesting to investigate the physiological role of mTOR in the individual cells of the inner ear. Such studies might include the investigation of cell type-specific mTORC1 or mTORC2 knock-out models.

As reviewed earlier, mTORC1 seems to play a role in auditory hair cell regeneration, while the role of mTORC2 in regeneration is less clear (see Section 3). How does mTOR interact with other signaling pathways? Is mTOR activation sufficient for hair cell regeneration? Which are the other mTOR-independent factors? Answers to these questions might open new therapeutic strategies to regenerate hair cells. Given the simultaneous role of mTORC1 in both auditory hair cell regeneration and the promotion of hair cell death, potential interventions must balance the beneficial effects of the former against the detrimental effects of the latter. In order to achieve this, future studies will need to precisely decipher the role of mTOR signaling in the inner ear.

## 6. Conclusions and Future Perspectives

Understanding mTOR signaling in the inner ear has only recently started to emerge. It has been shown that mTOR is involved in regenerative processes by promoting the proliferation and trans-differentiation of supporting cells into hair cells. Moreover, mTOR signaling is involved in auditory hair cell death and survival mechanisms. Therefore, advanced examination of this pathway could help to develop effective therapeutic strategies to prevent hearing loss and restore lost hair cells. Discrimination between the negative role of mTORC1 in hair cell survival and the positive role in hair cell regeneration will allow precise fine-tuning of potential therapeutic approaches. As reviewed earlier, mTORC2 might have a protective role and promote hair cell survival. However, future studies will need to dissect the precise roles of mTOR signaling in the inner ear. We posit that mTOR signaling has therapeutic potential to treat hearing loss.

## Figures and Tables

**Table 1:** Overview of major mTORC1 signaling components discussed in the text and their corresponding functions.

Protein(s)/Protein Complex	mTORC1 Signaling Related Function	References
mTOR	Serine/threonine kinase that forms the catalytic subunit of mTORC1	[10,71,72,73]
mLST8	mTORC1 subunit	[14,15]
RAPTOR	Defining and essential subunit of mTORC1	[15,16]
FKBP12	Forms a complex with rapamycin and inhibits mTORC1	[17,18]
AKT	Inhibits the TSC upon growth factor signaling	[23,24,25]
TSC1 TSC2 TBC1D7	Form the TSC which has GAP activity towards RHEB and therefore inhibits mTORC1	[26,28]
RHEB	GTP-bound RHEB activates mTORC1 upon growth factor signaling	[28,29]
RAG proteins	Signal amino acid availability to mTORC1	[31,32]
RAGULATOR complex	Binds the RAGs and is involved in amino acid signaling to mTORC1	[33]
GATOR1 complex	Negatively regulates RAGs to inhibit mTORC1	[36]
GATOR2 complex	Negatively regulates GATOR1 to activate mTORC1	[36]
KICSTOR complex	Translocates GATOR1 to the lysosome to negatively regulate mTORC1	[38,39]
SESTRIN-2	Leucine sensor, inhibits mTORC1 by binding GATOR2 when leucine levels are low	[40]
CASTOR-1 CASTOR-2	Arginine sensors, inhibit mTORC1 by binding GATOR2 when arginine levels are low	[41]
FLCN	Amino acid sensor positively regulating mTORC1	[42,43]
SAMTOR	SAM (methionine) sensor, binds GATOR1 and KICSTOR to inhibit mTORC1 when SAM/methionine levels are low	[44]
v-ATPase	Lysosomal amino acid sensor	[45]
SLC38A9	Lysosomal amino acid sensor	[46]
AMPK	Senses cellular energy status and inhibits mTORC1 by different mechanisms when cellular energy (ATP) is low	[12]
S6K	mTORC1 effector, is phosphorylated and activated by mTORC1 to promote anabolism	[10,59]
4E-BP	mTORC1 substrate, is phosphorylated and inhibited by mTORC1 to promote anabolism	[10,59,60]
LIPIN1	mTORC1 substrate, is phosphorylated and inhibited by mTORC1 to promote lipid synthesis	[53,61]
ATF4	Is activated by mTORC1 to promote nucleotide synthesis	[57]
HIF1α	Is activated by mTORC1 to promote glycolysis	[58]
ULK1	mTORC1 substrate, is phosphorylated and inhibited by mTORC1 to inhibit autophagy	[67,68,69,70]
ATG13	mTORC1 substrate, is phosphorylated and inhibited by mTORC1 to inhibit autophagy	[67,68,69]
TFEB	mTORC1 substrate, is phosphorylated and inhibited by mTORC1 to inhibit autophagy and lysosomal biogenesis	[12,54,55,56]

**Table 2:** Overview of major mTORC2 signaling components discussed in the text and their corresponding functions.

Protein	mTORC2 Signaling Related Function	References
mTOR	Serine/threonine kinase that forms the catalytic subunit of mTORC2	[10,71,72,73]
mLST8	mTORC2 subunit	[14,15]
mSIN1	mTORC2 subunit	[77,78,79]
RICTOR	Defining and essential subunit of mTORC2	[74,76]
AKT	mTORC2 effector, phosphorylated and activated by mTORC2	[92,93,94]
PKC	mTORC2 effector, phosphorylated and activated by mTORC2, modulates the actin cytoskeleton	[15,74,76]
SGK1	mTORC2 effector, phosphorylated and activated by mTORC2	[95]
SLC7A11	mTORC2 substrate, phosphorylated and inhibited by mTORC2	[96]
